# Plasticity of differentiated cells in wound repair and tumorigenesis, part II: skin and intestine

**DOI:** 10.1242/dmm.035071

**Published:** 2018-08-31

**Authors:** Joseph Burclaff, Jason C. Mills

**Affiliations:** Division of Gastroenterology, Departments of Medicine, Pathology & Immunology, and Developmental Biology, Washington University, St Louis, MO 63110, USA

**Keywords:** Dedifferentiation, Paligenosis, Plasticity, Regeneration, Stem cells, Tumorigenesis

## Abstract

Recent studies have identified and begun to characterize the roles of regenerative cellular plasticity in many organs. In Part I of our two-part Review, we discussed how cells reprogram following injury to the stomach and pancreas. We introduced the concept of a conserved cellular program, much like those governing division and death, which may allow mature cells to become regenerative. This program, paligenosis, is likely necessary to help organs repair the numerous injuries they face over the lifetime of an organism; however, we also postulated that rounds of paligenosis and redifferentiation may allow long-lived cells to accumulate and store oncogenic mutations, and could thereby contribute to tumorigenesis. We have termed the model wherein differentiated cells can store mutations and then unmask them upon cell cycle re-entry the ‘cyclical hit’ model of tumorigenesis. In the present Review (Part II), we discuss these concepts, and cell plasticity as a whole, in the skin and intestine. Although differentiation and repair are arguably more thoroughly studied in skin and intestine than in stomach and pancreas, it is less clear how mature skin and intestinal cells contribute to tumorigenesis. Moreover, we conclude our Review by discussing plasticity in all four organs, and look for conserved mechanisms and concepts that might help advance our knowledge of tumor formation and advance the development of therapies for treating or preventing cancers that might be shared across multiple organs.

## Abstract (precis)

**Summary:** This final installment of a two-part Review discusses how cycles of dedifferentiation and redifferentiation can promote tumorigenesis in the skin and intestine, showing how plasticity in these continuously renewing tissues might contribute to tumorigenesis.

## Introduction

**Fig. 1.**
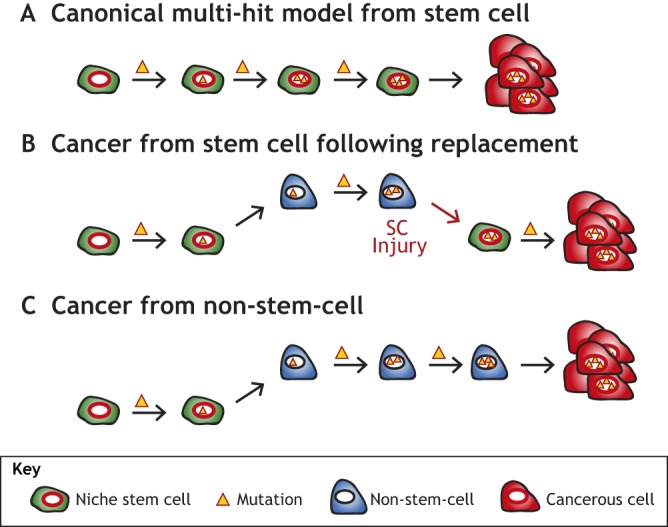
**Possible sources of tumor cells of origin.** (A) The canonical multi-hit model of tumorigenesis posits that stem cells (SCs) accumulate and store the necessary mutations for tumors to initiate. The tumor would arise from the normal SC niche and would not necessitate plasticity at any point to occur. (B) Experiments in many tissues have demonstrated that SCs can be replaced by more differentiated cells that revert to SCs and re-enter the niche. This allows for mutations needed for tumor initiation to be acquired and stored while the cell is in a non-SC fate (i.e. label-retaining cell, committed progenitor, differentiated cell, etc.). This would appear as tumors arising from the normal SC niche, yet would still incorporate earlier plasticity only visible through careful tracking. (C) Tumors may also arise from non-SC populations which never fully revert to a SC fate and re-enter the niche. Tumors arising directly from mature cells would originate in a tissue area outside of the normal SC niche.

In Part I of this Review (Burclaff and Mills, 2018), we discussed how long-lived, largely post-mitotic secretory cells in the stomach and pancreas can reprogram to re-enter the cell cycle after injury following a seemingly remarkably conserved process that we have termed paligenosis (Willet et al., 2018). We proposed that an unfortunate consequence of long-lived cells having the potential to undergo rounds of paligenosis and redifferentiation is that they might accumulate and store mutations until a final tumor-initiating mutation induces a dysplastic change that locks cells in a proliferative, pre-cancerous state (Fig. 1C). We have proposed this as the ‘cyclical hit’ model of tumorigenesis (Mills and Sansom, 2015; Saenz and Mills, 2018) and suggested that mature long-lived cells should be considered as potential tumor cells of origin. Historically, the search for which normal cells give rise to cancer has focused largely on tissue stem cells (SCs) because they are the most replicative, and rapid cell division correlates with an increased risk for acquiring mutations (Fig. 1A). Differentiated cells had not been thought to be involved in initiating tumorigenesis (White and Lowry, 2015). However, here we discuss how recent data implicating differentiated cells as contributors to cancer may help explain: (1) how SCs, now considered relatively short-lived in some tissues, can accumulate mutations over decades, i.e. mutations can be stored in differentiated cells that are recruited back into the SC state; (2) how organs without constitutive SCs (such as the pancreas) might acquire cancer; and (3) why genome sequencing often reveals numerous mutations in seemingly normal differentiated cells surrounding the cancer cells, i.e. ‘normal’ cells may accumulate mutations over decades of cyclical hits until one clone undergoes paligenosis to re-enter the cell cycle and spawn a tumor.

In Part II of this Review, we expand our discussion of plasticity and tumorigenesis to two well-studied, diverse organs: the skin and intestines. Both have intricate and dynamic SC hierarchies and undergo continuous full-tissue turnover, yet they differ in structure, function, developmental origin and cell types. Recent studies have uncovered numerous plastic events occurring in both organs, including intricate interconversions (Box 1) among various SC populations and dedifferentiation of mature cell types to a progenitor-like or even embryonic-like state. The well-defined cell types and array of lineage-tracing (Box 1) markers available within these organs have pointed to plasticity at multiple levels. Thus, our picture of cell interconversions and individual cell ontogenies in these organs is somewhat at a ‘higher resolution’ than in the pancreas and stomach (as discussed in Burclaff and Mills, 2018). For example, in the intestines, multiple molecular markers have identified possible progenitor and quiescent SC (qSC) populations that can replace the constitutive Lgr5^+^ SC, which themselves can be studied by several markers and promoter tools, following injury. The availability of these molecular tools in the skin and intestine has resulted in intricate experiments that support a ‘cyclical hit’ model of tumorigenesis. In this model, cells that carry mutations have left the SC niche to become progenitors, qSCs or more differentiated cells, but can be recalled to the SC niche (see Box 1 for a glossary of terms) following damage, introducing potentially tumorigenic mutations into the SC niche (Fig. 1B). Because SCs can arise from more differentiated cells, even tumors derived directly from niche-residing SCs may have actually depended on the plasticity of non-SCs ancestors of the current niche-residing SC at one point in their development. In short, here we discuss how comparing potentially conserved mechanisms for cellular plasticity among different organ systems may uncover additional nuance and perspective on how regeneration and tumorigenesis occur.

**Box 1. Glossary**

**APC:** adenomatous polyposis coli protein. Negatively regulates β-catenin-mediated Wnt signaling.

**β-catenin:** predominantly a cytoskeleton-associated protein that can also relocate to the nucleus to transduce Wnt signaling.

**Crestin:** a marker normally seen in embryonic neural crest cells of zebrafish. It is re-expressed in dedifferentiated melanocytes preceding melanoma formation.

**Crypt:** also known as crypt of Lieberkühn. The deepest, invaginated, portion of the small intestine and colonic epithelium, where all homeostatic intestinal proliferation occurs.

**Dermal papilla:** small bud of the uppermost layer of the dermis; extends into the base of the hair follicle to provide nourishment and signaling molecules.

**Ectopic:** in an abnormal location.

**Hedgehog signaling:** a cellular signaling pathway involved in differentiation. Hedgehog (Hh) ligands are received by the Patched receptor, which allows Smoothened to accumulate and modulate downstream transcription factors.

**Interconversion:** plasticity involving one stem cell population switching their identity to become a separate stem cell population.

**Intravital microscopy:** microscopy in living animals allowing for observation of biological processes *in vivo*. Also known as ‘live imaging’.

**Label-retention assays:** experimental techniques that use radioactivity or other tracers to mark the DNA of cells. The label becomes diluted as cells divide and disperse their DNA between daughter cells. Label-retaining cells (LRCs) maintain their labeling for extended periods of time, indicating that they divided at least once to incorporate label, but did not divide frequently thereafter, so their DNA retained the label, which indicates that the cells are slowly dividing.

**Lineage tracing:** experiments to determine all progeny from a specific cell. Uses cell-specific promoter genes to express reporter genes in target cells and their progeny.

**Melanocyte stem cells:** stem cells that originate from neural crest cells that migrate into the bulge during development and give rise to mature melanocytes that generate melanin (pigment), generally for the hair and skin.

**Nude mice:** mice that have an inhibited immune system as they are congenitally athymic and therefore produce a greatly reduced number of T cells.

**Ras** **superfamily:** a gene family encoding for small GTPase proteins that transmit signals when activated, often promoting genes involved in cell growth and survival. *HRAS*, *KRAS* and *NRAS* are commonly mutated in human cancers (Downward, 2003).

**Sebaceous gland:** a small gland attached to the top of the hair follicle containing lipid-rich, sebum-producing sebocytes to lubricate the skin and hair.

**Stem** **cell niche:** an area of tissue in which stem cells reside and which provides the necessary nutrients and signals to keep them in an undifferentiated and self-renewing state.

**Suprabasal:** above the basal layer. In the interfollicular epidermis, this term implies that the cell is differentiated, not a basal stem cell or progenitor cell.

**Transit amplifying (TA) cells:** rapidly proliferating cells with limited potential to give rise to other cell types, i.e. they produce daughter cells for differentiation but cannot self-renew more than a few times. TA cells are found in hair follicles, intestinal crypts and hematopoietic niches.

**Two-photon live imaging:** the use of two-photon microscopy in living organisms (e.g. mice), allowing for live imaging of tissue up to 1 mm in depth.

**Villi:** epithelial projections extending into the intestinal cavity. Intestinal villi maximize the surface area of nutrient-absorbing enterocytes.

**Wnt signaling:** a signaling pathway controlling cell fate and proliferation, among other processes. Wnt ligands are bound by the Frizzled receptor, which in turn stops a complex containing APC from degrading β-catenin. If free (non-cytoskeleton-associated) β-catenin accumulates, it relocates to the nucleus to coordinate gene transcription events characteristic of the Wnt response. Thus, deficient APC or constitutively active β-catenin potentiate the transcriptional output of active Wnt signaling.

**Xenografts:** tissue or tumor transplanted from a donor to a host of a different species, i.e. human tumor cells transplanted into a mouse.

## Skin

**Fig. 2.**
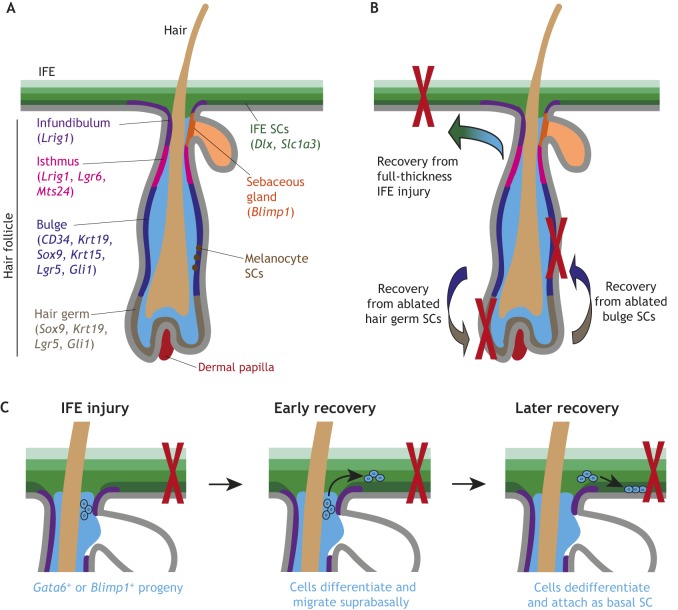
**The hair follicle (HF) and its responses to injury.** (A) The healthy HF has many distinct compartments that each have stem cell (SC) populations at the basal layer. These include the infundibulum at the top, the sebaceous gland, the isthmus, the bulge, and the hair germ at the bottom. (B) Following full-thickness interfollicular epidermis (IFE) injury, cells from the HF aid in IFE recovery, even becoming long-lived IFE stem cells. Also, if the bulge or hair germ is laser ablated, the remaining cells can interconvert to replace the missing cell populations. (C) Following epidermal injury, progeny from *Gata6*^+^ or *Blimp1*^+^ sebaceous gland SCs exit the HF to aid in recovery. The cells migrate to the wound bed suprabasally, then dedifferentiate and reattach to the basement membrane to act as SCs. CD34, cluster of differentiation 34; *Krt*, keratin; *Sox9*, SRY-box 9; *Lgr5/6*, leucine-rich repeat-containing G-protein coupled receptor 5/6; *Gli1*, glioma-associated oncogene homolog 1; *Blimp1*, B lymphocyte-induced maturation protein 1; *Lrig1*, leucine-rich repeats and immunoglobulin-like domains 1; *mts24*, antibody designation; *Dlx1*, distal-less homeobox 1; *Slc1a3*, solute carrier family 1 member 3.

The skin is the largest organ in the body, primarily consisting of the interfollicular epidermis (IFE) with hair follicles (HFs) as one of the major appendages. Early work in the skin found proliferating cells along the IFE basement membrane (BM) (Pinkus, 1952) and in the HF matrix (Van Scott and Ekel, 1958). Christopher Potten later used label-retention assays (Box 1) to show that slower-proliferating SCs are surrounded by quickly proliferating progenitors in the basal IFE (Potten, 1974), which improved our understanding of the skin SC and progenitor populations. Similarly, Cotsarelis discovered label-retaining SCs along the outer wall (bulge) of the HF (Cotsarelis et al., 1990). It took another decade to prove that these HF-SCs were multipotent and able to generate all lineages within the skin using early lineage-tracing techniques (Oshima et al., 2001). It is now known that there are at least two distinct IFE SCs populations (Table 1) (Sada et al., 2016), with their progeny rising through the epidermal layers of the stratified squamous epithelium as they differentiate (Fuchs and Raghavan, 2002; Clayton et al., 2007). Further lineage-tracing studies have shown that the HF and IFE normally derive from functionally distinct SC populations (Ghazizadeh and Taichman, 2001; Levy et al., 2005) and there is additional SC diversity within the distinct HF compartments (Jaks et al., 2010) (Fig. 2A). SCs within the HF bulge were first functionally determined using histone-2B label retention (Box 1) (Tumbar et al., 2004) and later found to express several distinctive markers (Table 1). Progeny from these SCs move off the BM and into the follicle matrix to become transit amplifying (TA) cells (Box 1). Melanocyte SCs (Box 1) also reside in the bulge and give rise to mature melanocytes, which migrate to the lower HF or the IFE (Mort et al., 2015). At the bottom of the follicle, the hair germ maintains distinct SCs that regenerate the follicle upon hair loss (Ito et al., 2004). Growth signals from the mesenchymal dermal papilla (Box 1) at the bottom of the HF are necessary for proper bulge cell proliferation, (Greco et al., 2009; Rompolas et al., 2012), although loss of dermal papilla can be experimentally rescued by activation of β-catenin (Box 1) within the SCs (Deschene et al., 2014). When transplanted, dermal papilla cells are sufficient to induce new HF formation and growth within the epidermis (Oliver, 1970; Jahoda et al., 1984), which can also be partially recapitulated with activated β-catenin (Gat et al., 1998). Above the bulge lies the sebaceous gland (Box 1), with B lymphocyte-induced maturation protein 1 (*Blimp1*)*^+^* progenitors (Horsley et al., 2006) maintaining the sebocyte (Schneider and Paus, 2010) population. The upper ridge of the HF, the infundibulum, is maintained in part by cells with elevated expression of leucine-rich repeats and immunoglobulin-like domains 1 (*Lrig1*) that also contribute to the sebaceous gland (Jensen et al., 2009; Page et al., 2013). Finally, the isthmus between the upper bulge and the sebaceous gland houses heterogeneous progenitors characterized by an array of markers (Table 1).

**Table 1.**
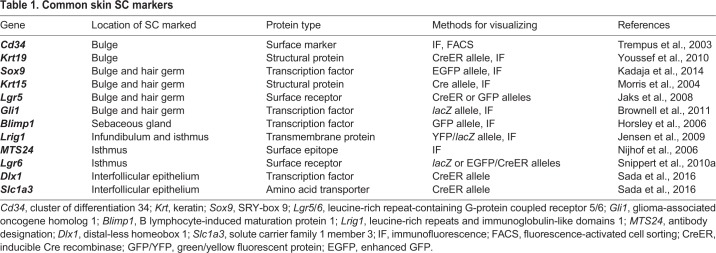
**Common skin SC markers**

### Plasticity in the skin

The various regions in each HF compartment had largely been studied in isolation, yet it is now clear that the progenitor and differentiated cells are capable of considerable plasticity. HF SCs continuously change proliferative state throughout the hair-cycle phases of anagen (growth), catagen (retraction) and telogen (rest) (Müller-Röver et al., 2001; Fuchs, 2009). HF SCs convert between these active and quiescent states based on their distance from the dermal papilla (Greco et al., 2009), with dynamic chromatin remodeling regulating their ability to transition between phases while maintaining cell identity (Lien et al., 2011).

The recent advent of intravital microscopy (Box 1) has greatly facilitated skin plasticity studies (Park et al., 2016). Although intravital imaging can be used in internal organs such as the intestine (Ritsma et al., 2014), the skin is far more accessible, allowing for rapid advances in our knowledge of skin-cell population dynamics. Greco and coworkers pioneered two-photon live imaging (Box 1) (Rompolas et al., 2012) to demonstrate that populations of SCs residing in different locations of the HF are capable of interconverting upon injury: if the bulge or hair germ is laser ablated, the remaining niche can repopulate the lost SCs and regain full function (Rompolas et al., 2013) (Fig. 2B). This fits with another study showing that CD34^+^ SCs in the upper bulge are able to replace *Lgr5^+^* SCs in the lower bulge and hair germ that are lost upon targeted ablation (Hoeck et al., 2017). Similarly, the discrete IFE SC populations can interconvert if one of them is ablated (Sada et al., 2016).

As in other organs, injury changes the proliferative dynamics in the skin (Donati and Watt, 2015). HF and IFE SCs maintain distinct cellular populations at homeostasis (Levy et al., 2005). Upon epidermal wounding, IFE SCs drive much of the regeneration (Mascré et al., 2012), but follicular cells also aid in repopulating the IFE, with cells from nearly all HF compartments streaming into the wound bed (Ito et al., 2005; Page et al., 2013; Goodell et al., 2015). Many of these cells are short-lived and are quickly replaced by IFE cells, yet some HF-originating cells reprogram into long-lived IFE progenitors following wounding, although it is unclear whether these cells were originally SCs or more differentiated progeny (Levy et al., 2007) (Fig. 2B). A recent study suggests that these changes are driven by chromatin rearrangements within the SCs, which override the normal SC homeostatic enhancers (Ge et al., 2017).

The dynamics of the migration to the wound bed have long been debated (Headon, 2017). Skin may heal with a ‘wavefront’ model, with cells migrating into the wound bed being led by the basal progenitors (Radice, 1980; Safferling et al., 2013), or it could use a ‘leapfrog’ model with migration led by early differentiated cells detaching from the BM, migrating, then dedifferentiating and re-attaching to the BM at the wound bed (Krawczyk, 1971; Paladini et al., 1996). Skin cells have long been considered unable to dedifferentiate, supporting the wavefront model, and two recent studies reinforce this conclusion (Aragona et al., 2017; Park et al., 2017). However, another recent study from the Watt lab demonstrates that, upon IFE puncture, *Gata6*^+^ cells from the sebaceous gland migrate out from the HF suprabasally (Box 1) then dedifferentiate and reattach to the BM at the wound site as SCs (Fig. 2C) (Donati et al., 2017). The authors saw similar dedifferentiation of *Blimp1^+^* progenitors as well, leading to the speculation that ‘dedifferentiation may be a general property of terminally differentiated epidermal cells following wounding’ (Donati et al., 2017). It will be interesting to determine whether skin cell dedifferentiation follows the conserved paligenosis pattern involving early autophagy and dynamic mammalian target of rapamycin complex 1 (mTORC1) regulation (Willet et al., 2018).

### Skin tumorigenesis

The skin presents interesting opportunities for studying tumorigenesis because it has been shown that phenotypically healthy aged human skin harbors large (up to multiple square millimeters) clones carrying numerous genomic mutations, including known driver mutations associated with squamous cell carcinoma (SCC; Box 2) (Martincorena et al., 2015). Studies in mice reinforce that skin cells can maintain mutations without forming tumors: expressing constitutively active *Kras*^G12D^ (see Ras superfamily, Box 1) in *Lrig1^+^* SCs at the infundibulum of the HF does not induce tumors, unless the epidermis is wounded with a biopsy punch (Page et al., 2013). This is similar to the finding that *Kras*^G12D^ is unable to drive pancreatic cancer without induced inflammation (Guerra et al., 2007), as discussed in Part I of this Review (Burclaff and Mills, 2018). This conserved need for wounding highlights how mutations can be stored in the cellular lineages long term, until some aspect of the recovery response, such as changes within the SCs or in their progeny, initiates tumor formation. Studies have shown that expression of numerous oncogenes in various skin SCs can initiate tumors (Brown et al., 1998; Youssef et al., 2010; Kasper et al., 2011; Wong and Reiter, 2011; Page et al., 2013), yet many studies have shown a requirement for multiple hits, such as deletion of transforming growth factor-beta receptor type 1 (*Tgfbr1*), replacing the effect of wounding to initiate rapid tumor formation from bulge SCs primed with mutations in the Ras pathway (Cammareri et al., 2016).

**Box 2. The main types of skin cancer**

The most common types of skin cancer are squamous cell carcinoma (SCC), basal cell carcinoma (BCC) and melanoma. They arise through varying molecular and cellular events and have different phenotypes and prognoses.

BCC and SCC account for the vast majority of non-melanoma skin cancer cases, and both arise from keratinocytes within the stratified squamous skin epithelium. BCC and SCC tumors differ in many ways. For one, grossly, BCC tumors appear smooth and translucent, whereas SCC tumors are rough or scaly and fast growing. BCCs also rarely metastasize, whereas SCC tumors can spread at a low but not insignificant rate (Cakir et al., 2012). Ultraviolet (UVB) irradiation from the sun promotes both tumor types (Gallagher et al., 2010), although studies have indicated that childhood sun exposure is more important for BCC, whereas SCC appears to be affected by total lifetime sun exposure (Rosso et al., 1996; Zak-Prelich et al., 2004). Common mutations also differ between the two. Over 70% of BCCs harbor mutations in Patched or Smoothened, implicating the Hedgehog signaling pathway (Xie et al., 1998), whereas SCC is more likely to exhibit mutations in p53 (Black and Ogg, 2003). BCC and SCC also arise through different events, with BCC tumors forming *de novo* from phenotypically undamaged skin and SCC arising from precursor lesions or damage (McGillis and Fein, 2004). The specific cells of origin for both remain unclear (Song and Balmain, 2015).

Melanomas are less frequent than SCC and BCC but also have the highest predilection for metastasis. Unlike keratinocyte carcinomas, melanomas arise from the melanocyte lineage, which originates from embryonic neural crest cells. Melanomas are also promoted by sun exposure, but genetic analysis indicates that UVA irradiation is more important for melanomas than UVB (Wang et al., 2001). Although it is clear that melanomas arise from cells within the melanocyte lineage, it remains unclear at which level of differentiation the cells of origin need to be to permit malignant transformation (Köhler et al., 2017; Moon et al., 2017).

There is also evidence that multiple skin cancers (Box 2) can arise through dedifferentiation (Song and Balmain, 2015). This was first demonstrated in 1990, when the Balmain lab expressed mutant *Hras* under the control of the keratin 10 (*Krt10*) promoter, whose expression they showed to be constrained to suprabasal IFE cells (i.e. more mature cells and not SCs). They supplied no experimental second ‘hit’, yet still observed SCC formation at sites of frequent wounding, such as the base of the tail and around the ear tags (Bailleul et al., 1990). Thus, wounding was also necessary to promote tumorigenesis in this system, akin to earlier experiments showing that repeated mechanical injury (cutting) is sufficient to promote tumor initiation in skin primed with a topical carcinogen (Förstenberger et al., 1989) and in agreement with our understanding of tumorigenesis in pancreas and stomach (Burclaff and Mills, 2018). Differentiated cells could also serve as cancer cells of origin when mutant *Hras* expression was forced under the regulation of the *Krt1* promoter, which drives expression in suprabasal cells or fate-determined, post-mitotic basal cells (Greenhalgh et al., 1993). No recent studies have replicated expressing oncogenes in *Krt1*^+^ or *Krt10*^+^ cells, as has been done for other skin SC populations, so the potential contribution from unintended *Hras* signaling in other cells in these original studies remains unknown. However, the concept of plasticity in mature skin cells is supported by recent results showing other suprabasal cells being recruited to act as basal IFE SCs following injury (Donati et al., 2017). Future experiments might target these populations with mutations other than those in Ras genes, which are relatively uncommon in skin cancers, and with better reporting techniques to ascertain whether mutations more specific to skin cancer (Box 2) can initiate tumors from non-SCs with or without wounding.

The Blanpain lab showed that another type of skin tumor, basal cell carcinoma (BCC; Box 2) arises via dedifferentiation. They activated Hedgehog signaling (Box 1) in long-lived IFE progenitor cells to induce BCC in adult mice and found that the tumor-initiating cells reprogrammed to a state resembling embryonic HF progenitors, with high Wnt signaling (Box 1) and characteristic expression of embryonic genes. They further confirmed that similarly elevated Wnt activity and embryonic gene expression are present in human BCC tissue samples (Youssef et al., 2012).

Several recent studies support a role for dedifferentiation in melanoma initiation (Box 2). The first, by Kaufman et al., observed that melanoma arises from cells that first dedifferentiate to an embryonic-like state. Zebrafish melanocytes with mutant, constitutively active rapidly accelerated fibrosarcoma B (*BRAF^V600E^*; Box 1) in a *p53*-null background become tumor-initiating cells only after dedifferentiating to a state resembling embryonic neural crest cells characterized by SRY-box 10 (*sox10*) and *crestin* (Box 1) expression (Kaufman et al., 2016).

Two recent back-to-back articles used mouse models to analyze how the melanocyte lineage gives rise to melanomas. Moon et al. found that quiescent melanocyte SCs (MCSCs) in the HF bulge were refractory to *Braf*^V600E^-driven tumorigenesis and depletion of phosphatase and tensin homolog (*Pten*; Box 1), whereas melanomas arose within the same genetic background following MCSC activation by ultraviolet (UVB) radiation or drug-induced inflammation (Moon et al., 2017). The cellular dynamics were further analyzed by Köhler et al., who tracked the melanocytes shortly after activation and found that MCSCs needed to differentiate and migrate to the lower HF before being able to proliferate and initiate tumors, with no increased proliferation seen directly from the bulge MCSCs (Köhler et al., 2017). Further experiments using tumorigenesis models in the mouse tail skin, which has features that more closely mimic melanocyte location in human melanoma, again showed that mature melanin-producing melanocytes could initiate melanoma, while less mature amelanotic melanocytes were refractory to the mutations. Finally, a third study described that cultured mature melanocytes could be transformed into cancer-initiating cells by overexpressing Fos-related antigen 1 (*Fosl1*). These cells formed melanomas when injected into nude mice (Box 1; Maurus et al., 2017), proving that such reprogramming can occur, at least *in vitro*. Together, these studies demonstrate that mature melanocytes may act as cells of origin for melanoma via dedifferentiation, and add to the expanding literature indicating that dedifferentiation and plasticity may play a key role in initiating multiple skin cancers.

## Intestines

**Fig. 3.**
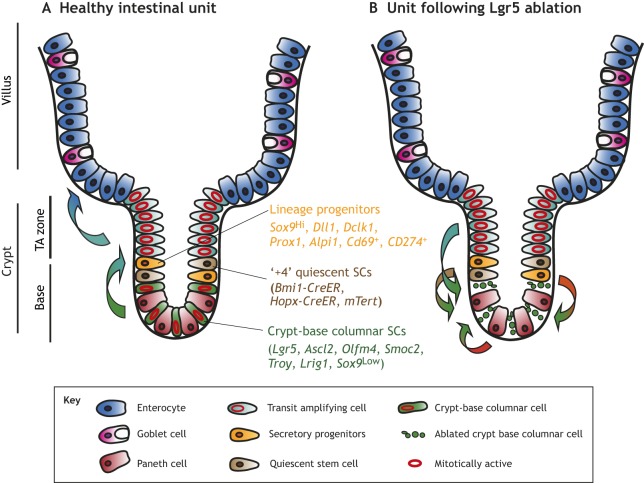
**The** **intestinal crypt and its response to stem cell (SC) loss.** The healthy intestinal unit, with SCs and Paneth cells (red) at the base, quiescent SCs (qSCs) and label-retaining cells (LRCs) directly above those, then transit amplifying (TA) cells (teal). Above the crypt are mature enterocytes (blue) and goblet cells (pink). Not shown: tuft cells and endocrine cells. Most proliferation occurs in the TA zone, with some at the crypt-base columnar (CBC) cells (green) and infrequent proliferation of the qSCs/LRCs. (B) Many cells can replace the basal SC population following ablation, including interconversion of qSCs (brown) and dedifferentiation of Paneth cell precursors, enteroendocrine cell precursors, goblet cell precursors, secretory progenitors and enterocyte progenitors (within the yellow population). *Lgr5*, leucine-rich repeat-containing G-protein coupled receptor 5; *Ascl2*, achaete-scute family BHLH transcription factor 1; *Olfm4*, olfactomedin 4; *Smoc2*, SPARC-related modular calcium binding 2; *Troy*, TNF receptor superfamily member 19; *Lrig1*, leucine-rich repeats and immunoglobulin-like domains 1; *Sox9*, SRY-box 9; *Bmi1*, B lymphoma Mo-MLV insertion region 1 homolog; *Hopx*, HOP homeobox; *mTert*, mouse telomerase reverse transcriptase; *Prox1*, prospero homeobox 1; *Dll1*, delta-like ligand 1; *Alpi1*, alkaline phosphatase, intestinal 1; *Dclk1*, doublecortin-like kinase 1; CreER, inducible Cre recombinase.

The intestines broadly comprise two principal histoanatomical organizational patterns: (1) the small intestine, with crypts (Box 1) extending towards the muscular wall, and villi (Box 1) extending into the lumen; and (2) the large intestine, with a flat surface and similarly invaginating crypts. Throughout, proliferation is confined to the lower portions of the crypts, and most progeny move up and out of the crypt as they differentiate, eventually sloughing off and being replaced. Early studies found that the proliferating cells at the crypt base include multipotent SCs able to produce all of the intestinal cell lineages (Cheng and Leblond, 1974). The discovery that *Lgr5* expression marks these crypt-base columnar (CBC) SCs (Barker et al., 2007) led to a rush of molecular work on intestinal cell fate in the past decade. CBC cells reside in a niche that includes epithelial Paneth cells and *Foxl1*^+^ mesenchymal cells beneath the BM that support division and repress differentiation (Sato et al., 2011; Roth et al., 2012; Aoki et al., 2016; Shoshkes-Carmel et al., 2018). CBC daughters exit the crypt base and rise into the TA zone in the middle/upper portion of the crypt, where the highest rates of proliferation occur. Out of the milieu of stem and TA cells arise the (largely) post-mitotic intestinal cell lineages: absorptive enterocytes, antimicrobial-secreting Paneth cells, mucus-secreting goblet cells, inflammation-coordinating tuft cells, and various populations of enteroendocrine cells (Fig. 3A).

In addition to active CBC and TA cells, early studies also indicated the existence of more slowly proliferating cells that survive to restore intestinal crypts after radiation-induced death of the faster-cycling populations (Hendry and Potten, 1974; Potten et al., 1974). These cells were characterized as long lived and rarely dividing, and were thus called label-retaining cells (LRCs) (Box 1). Later experiments indicated that these were qSCs, which rarely proliferate but are induced to do so upon injury (Li and Clevers, 2010). Many studies have used molecular markers and lineage tracing to characterize qSC populations residing around the +4 cell position above the basal-most crypt cell and below the TA cells (Fig. 3A). These label-retaining qSCs were originally considered to be the ‘true’ intestinal SCs: they were believed to be mostly quiescent and divide only rarely and always asymmetrically to produce one SC daughter and one daughter that would differentiate to other lineages (Potten et al., 1997).

There have been obstacles in definitively identifying these LRCs and qSCs (Smith et al., 2016; Yousefi et al., 2017), including the caveat that some transcripts expressed from gene promoters used to mark qSCs [mouse telomerase (*mTert*)-GFP, B lymphoma Mo-MLV insertion region 1 homolog (*Bmi1*)-CreER and HOP homeobox (*Hopx*)-CreER] can be detected in all basal cells (Itzkovitz et al., 2011; Muñoz et al., 2012; Li et al., 2014, 2016). Additionally, some genes have been studied using different promoter elements and mouse genetic approaches with divergent expression patterns, such as *Bmi1-CreER* and *Bmi1-EGFP* (Li et al., 2014), leading to conflicting results. Our current understanding of intestinal qSCs is that they are distinct from LRCs, with qSCs remaining in the quiescent G0 phase of the cell cycle (Li et al., 2016; Yousefi et al., 2016), allowing for rapid cell cycle re-entry following injury, whereas LRCs arrest in G1 and are suggested by some to be primarily Paneth cells (Li et al., 2016). Several markers for qSCs have been proposed, all of which are present in cells near the +4 crypt position, but some appear to mark multiple distinct populations (Table 2). qSCs occasionally give rise to CBC cells at homeostasis and more frequently upon tissue injury, yet it is unclear whether CBC cells commonly become qSCs (Li et al., 2014; Yousefi et al., 2017). Intestinal SC populations can also be distinguished based on their radiosensitivity: qISCs are largely radioresistant, perhaps owing to their arrest in G0 (Montgomery et al., 2011; Yan et al., 2012; Li et al., 2016), while mitotically active CBC cells are easily killed with radiation (Yan et al., 2012; Tao et al., 2015), suggesting differential sensitivity to DNA damage. The next section discusses how dichotomous sensitivity to DNA damage may be a key feature of plasticity within the intestines.

**Table 2.**
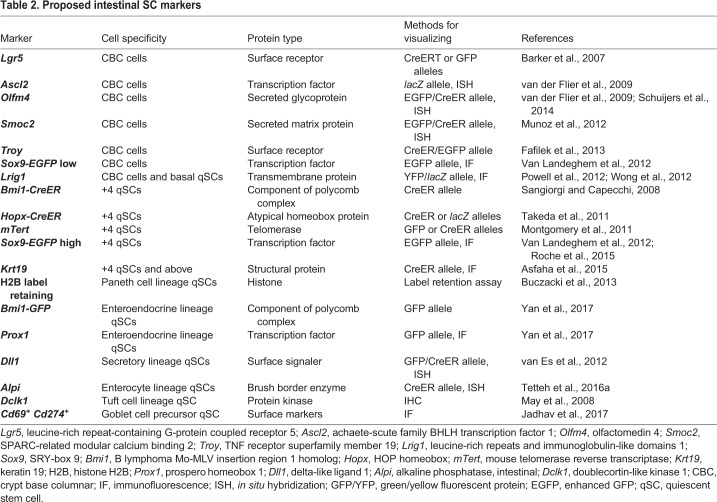
**Proposed intestinal SC markers**

### Intestinal plasticity

The interconversion between qSCs, marked by reporters driven by *Bmi1-CreER* (Sangiorgi and Capecchi, 2008) or *Hopx-CreER* (Takeda et al., 2011), and CBC cells has been demonstrated at homeostasis, where lineage tracing revealed that qSC progeny can give rise to all intestinal cells. In 2011, de Sauvage and coworkers showed that, upon ablation, CBC cells can be replaced by *Bmi1-CreER*-expressing qSCs (Tian et al., 2011). As mentioned above, it is the *Bmi1* transgene that specifically marks such cells, not the endogenous mRNA or protein product (Muñoz et al., 2012; Li et al., 2014). Numerous cell populations in various differentiation states were subsequently shown to dedifferentiate to replace the CBC cells following targeted ablation or irradiation (Fig. 3B). These include the secretory lineage and/or Paneth cell precursors (van Es et al., 2012; Buczacki et al., 2013; Roche et al., 2015; Jadhav et al., 2017), enteroendocrine cell precursors (Buczacki et al., 2013; Jadhav et al., 2017; Yan et al., 2017), and absorptive enterocyte progenitors (Tetteh et al., 2016a). However, targeted genetic ablation of *Lgr5^+^* CBC cells with concurrent irradiation causes extensive intestinal atrophy with reduced regeneration (Metcalfe et al., 2014). These results are consistent with two possible interpretations: that some portion of *Lgr5*-expressing cells may survive irradiation and become integral for the regenerative response (Metcalfe et al., 2014), or that the cells that dedifferentiate to replace lost CBC cells rapidly express enough *Lgr5* to be targeted, and therefore ablated, themselves. The mechanism for recruiting qSCs via dedifferentiation is not well defined, yet yes-associated protein 1 (*Yap1*) is known to be required for proper regeneration following irradiation (Gregorieff et al., 2015). The Lengner lab demonstrated that Musashi (MSI) RNA-binding proteins and mTORC1 activity are necessary and sufficient for qSCs to re-enter the cell cycle (Yousefi et al., 2016, 2018). The latter finding is particularly interesting given the key role that mTORC1 plays in paligenotic recruitment of mature gastric and pancreatic cells to a proliferative state (Willet et al., 2018).

In addition to the aforementioned plasticity of qSCs and progenitors, a recent report shows that irradiation can revert mature Paneth cells to a proliferative state in which they give rise to other intestinal lineages via a Notch1-mediated mechanism (Yu et al., 2018). Paneth cells are close relatives to gastric chief cells and pancreatic acinar cells (Burclaff and Mills, 2018), with all three being large, long-lived, normally non-proliferative secretory cells that express the transcription factor *Mist1* (Lo et al., 2017). The authors show that *Yap1* is upregulated in these Paneth cells as they dedifferentiate, although it has not yet been shown whether they undergo the stages of paligenosis as seen in their gastric and pancreatic counterparts (Willet et al., 2018).

### Intestinal tumorigenesis

As with many adult cancers, the cell of origin for intestinal tumors is actively debated (Huels and Sansom, 2015). Human intestinal tumors frequently occur in a setting of adenomatous polyposis coli (APC; Box 1) loss, active Wnt signaling and KRAS activation (Lamlum et al., 2000). Most experimental tumorigenesis models recapitulate those aberrations or express constitutively active β-catenin to induce canonical Wnt signaling (Harada et al., 1999). The canonical multi-hit theory (Vogelstein and Kinzler, 1993) describes intestinal SCs as the cells of origin, as they were considered a long-lived proliferative population that could thus give rise to tumors (Fearon and Vogelstein, 1990; Vogelstein and Kinzler, 1993). Consistent with this, ‘bottom up’ tumorigenesis, i.e. originating from the crypt base, often occurs in humans, with the earliest neoplastic cells seemingly originating from the crypt (Preston et al., 2003). Similarly, APC deletion or expression of stabilized β-catenin in CBC cells is sufficient for tumor initiation in mice (Barker et al., 2009; Zhu et al., 2009). Mouse studies have demonstrated that the CBC cells are not alone in their tumor-generating capacity: constitutive β-catenin activation in *Bmi1-CreER* qSCs (Sangiorgi and Capecchi, 2008) or APC deletion in *Krt19*^+^ qSCs (Asfaha et al., 2015) were both sufficient for tumorigenesis.

It should be noted that SCs rapidly give rise to other intestinal epithelial cell types, so even though a tumor may arise in a scenario where an oncogene is expressed in an SC, that doesn't necessarily mean that the last non-neoplastic cell before transition to cancer was the SC itself. Thus, while the physical phenomenon of tumors originating in crypts (i.e. ‘bottom up’) clearly occurs in both humans and mice, it is currently difficult to ascertain whether such tumors arise directly from the CBC cell itself or from its more differentiated progeny. Moreover, as we know that numerous populations can replace the CBC cell, it is possible that basal tumors might originate from more differentiated daughters that regress back into the crypt base after garnering neoplastic mutations (Fig. 1B).

Consistent with the role for plasticity in tumorigenesis in other organs, evidence suggests that intestinal tumors can arise from post-mitotic cells residing above the proliferative crypt, which dedifferentiate and re-enter the cell cycle. ‘Top down’ tumorigenesis (Shih et al., 2001) was noted in humans half a century ago, with tumors observed at the tops of colon crypts detached from the base (Cole and McKalen, 1963), although these were often attributed to cutting artifacts or proliferating cells migrating from the crypt base (Maskens, 1979). Mouse models later confirmed that differentiated cells of the small intestine and colon can cause tumors in experimental settings.

Injury and inflammation, specifically activated nuclear factor kappa-light-chain-enhancer of activated B cells (NF-κB) signaling, are commonly associated with tumors, and two early experiments used inflammation to induce tumorigenesis from non-proliferative intestinal cells. In one such study, mature enterocytes formed tumors on the villi when β-catenin and NF-κB were specifically activated in them using the X-box-binding protein 1 (*XBP1*) promoter (Schwitalla et al., 2013). In a parallel report, long-lived colonic tuft cells lacking APC could form tumors only when treated with the inflammatory agent dextran sodium sulfate (DSS) (Westphalen et al., 2014). In yet another example where dedifferentiation might be key, Tetteh et al. bypassed inflammation and induced tumor formation in differentiated colon cells lacking APC by forcing mutant *Kras* expression (Tetteh et al., 2016b), demonstrating again that cells above the proliferative colonic crypt can initiate tumorigenesis. Thus, these experiments are similar to those described for skin, pancreas and stomach, wherein injury causes differentiated cells to dedifferentiate (i.e. undergo paligenosis) and unmask mutations as they re-renter the cell cycle that result in tumorigenesis.

Other experiments in mouse models indicate how the ability to maintain proper villus differentiation is important to avoid tumorigenesis. Bone morphogenic protein (BMP) signaling promotes normal crypt-villus differentiation, and expression of the BMP antagonists noggin (*Nog*) (Haramis et al., 2004) or gremlin 1 (*Grem1*) (Davis et al., 2015) in all intestinal cells via the villin-1 (*Vil1*) promoter blocks differentiation and induces tumor formation, with ectopic (Box 1) proliferative crypts forming on villi perpendicular to the normal crypt plane. The origin of these ectopic crypts is not clear, and they could arise via dedifferentiation or from expansion of otherwise normal crypt progenitors in an aberrant niche. Similar ectopic proliferative crypts were observed upon Hedgehog (Box 1) inhibition (Madison et al., 2005) or mesenchymal *Bmpr1a* knock out (Lim et al., 2017). Finally, a recent study from the Sansom lab demonstrates that mice lacking *APC* and *Tgfbr1* while expressing mutant *Kras*^G12D^ via the *Vil1* promoter experience both ‘bottom up’ and ‘top down’ tumorigenesis. They further show that MEK inhibition blocks tumorigenesis at the villi but not the base (Cammareri et al., 2017). This suggests that different mechanisms are likely involved in the different compartments, even though the resulting tumors have surprisingly similar genetic profiles. As MAPK signaling (downstream of MEK) is necessary for gastric and pancreatic paligenosis (Collins et al., 2014; Khurana et al., 2013), these results might also indicate that blocking plasticity directly inhibits initiation of tumorigenesis in differentiated cells, an effect that might be replicable across organs.

Although there are multiple potential instances of tumors arising either directly from dedifferentiated (plastic) mature cells or from SCs that developed from dedifferentiating mature cells, all the studies have caveats. Some lack lineage tracing to affirm the molecular features of the tumor-initiating cells; many make inferences based on infrequent tumor events, hampering generalization; and, in all cases, either multiple mutations or tissue injury were required for tumor formation, obfuscating the proximate cells of origin for the tumors. However, in aggregate, the studies indicate that numerous cells aside from crypt SCs can act as tumor cells of origin. As in other organs, inflammation may induce cell plasticity, as is the case with DSS causing inflammation and dedifferentiation in the intestine, possibly mimicking how ulcerative colitis may increase the risk of colorectal cancer in patients (Eaden et al., 2001). In short, injury-induced intestinal plasticity may resemble the metaplasia/plasticity in spasmolytic polypeptide-expressing metaplasia in the stomach and acinar-to-ductal metaplasia in the pancreas [discussed in Part I of the Review (Burclaff and Mills, 2018)] or in injuries that promote skin tumors, as discussed above.

A role for plasticity has also been shown within established intestinal malignancies. *Lgr5*^Hi^ cells in tumors, defined by increased *Lgr5* transcript levels upon fluorescence-assisted cell sorting, are often thought to maintain the SC features of *Lgr5^+^* CBC cells and are frequently regarded as stem-like cells for these tumors (Merlos-Suárez et al., 2011). Cells with such properties have been termed cancer SCs (CSCs) (Barker et al., 2009; Schepers et al., 2012). Similar to intestinal CBC cells, recent studies demonstrate that *Lgr5*^Hi^ cells are not necessary for tumor maintenance. *Lgr5*^Hi^ cells in mouse colorectal tumors are replaceable upon targeted ablation, although, intriguingly, they are necessary for liver metastasis (de Sousa e Melo et al., 2017). Similar results occur upon ablation of *Lgr5*^Hi^ cells in human colorectal tumor xenografts (Box 1), where cells expressing differentiation markers such as keratin 20 (KRT20^+^) could regenerate the ablated *Lgr5*^Hi^ tumor cell population (Shimokawa et al., 2017). Even continued ablation of *Lgr5*^Hi^ cells in existing tumors using targeted antibody-drug conjugates resulted only in smaller tumors and longer animal survival, but not full recovery (Junttila et al., 2015). The ability of tumors to recover from acute *Lgr5*^Hi^ cell ablation and to slowly grow even with constitutive loss of *Lgr5*^Hi^ cells suggests that rounds of dedifferentiation may continue even after tumor formation, perhaps allowing for the accumulation of additional mutations that lead to advanced tumor grades, metastasis or acquired resistance to therapy.

## Conclusion

Plasticity is important in diverse organs, from the continuously regenerating luminal gastrointestinal tract to the non-proliferative pancreas (Burclaff and Mills, 2018) and the highly compartmentalized skin. It can manifest as focal interconversion of normally distinct SC populations that is largely undetected outside of careful lineage tracing, or as large-scale metaplasia and dedifferentiation of long-lived mature cells. Cellular plasticity thus seems a critical feature of tissue repair, but it is also clear that plasticity has the unfortunate side effect of allowing tissues additional means to accrue, store and eventually unmask oncogenic alterations that drive tumorigenesis.

In Part I of this Review, we discussed studies involving the stomach and pancreas that support the ‘cyclical hit’ model of tumorigenesis, with long-lived cells undergoing paligenosis and then redifferentiating in response to environmental stimuli, accumulating mutations until a final mutation locks them in a proliferative state (Fig. 1C) (Burclaff and Mills, 2018). Studies from the skin and intestine reviewed in the present article show that SCs are replaced upon targeted ablation (Hsu et al., 2011; Tian et al., 2011; Rompolas et al., 2013; Ritsma et al., 2014; Hoeck et al., 2017). Interestingly, this may also occur in the stomach during recovery of SC function following inhibition of proliferation (Radyk et al., 2018). Thus, various cellular populations can revert to a SC state following injury. The findings that even longer-lived, fully differentiated Paneth cells can be called back into the SC niche indicate how cycles of dedifferentiation and redifferentiation could lead to the eventual accumulation, storage and unmasking of mutations in the intestines (Yu et al., 2018), just as it might occur in the pancreas and stomach (Fig. 1B). In fact, the ‘cyclical hit’ model for mutation accumulation may be especially pertinent in the intestine, where past experiments using very low doses of radiation imply that cells at the crypt base undergo apoptosis in response to even slight perturbations to their genomic DNA (Ijiri and Potten, 1990; Potten and Booth, 1997; Potten, 1998). If CBC cells are truly highly sensitive to DNA damage, they may actually be unable to accrue mutations on their own, necessitating replacement by other cells for any new ‘hit’ to be accumulated. While intriguing, these older observations should be replicated with modern lineage-tracing techniques to monitor how sensitive to mutations the CBC cells (and possibly other SC populations) actually are.

The skin currently has less clear examples of mature cell plasticity leading to tumors than in gastrointestinal organs, yet the available evidence supports that it may occur. Multiple investigators have shown that melanocytes form melanomas via dedifferentiation, and many plasticity markers characteristic of the stomach, pancreas and intestine also become upregulated during this progression (Table 3). It will be interesting to see whether melanocytes undergo the three stages of paligenosis as seen in other dedifferentiating cell types. Two reports detail the activation of *Hras* in non-SC populations of the IFE driving tumor formation (Bailleul et al., 1990; Greenhalgh et al., 1993), but the authors did not have lineage-tracing tools available. It should be noted though, that, even with lineage tracing such as Cre-recombinase-based studies, unintentional recombination may occur in untargeted cells. It is thus imperative to carefully identify and track the labeled cells with multiple concomitant methods to help resolve the cell of origin in such studies (see a brief discussion of caveats of lineage tracing in Saenz and Mills, 2018). Some instances of apparent plasticity highlighted via lineage tracing may therefore simply be cases wherein a differentiated cell promoter was expressed in a SC, thereby making it appear as if there had been a conversion of a mature cell to a SC phenotype.

**Table 3.**
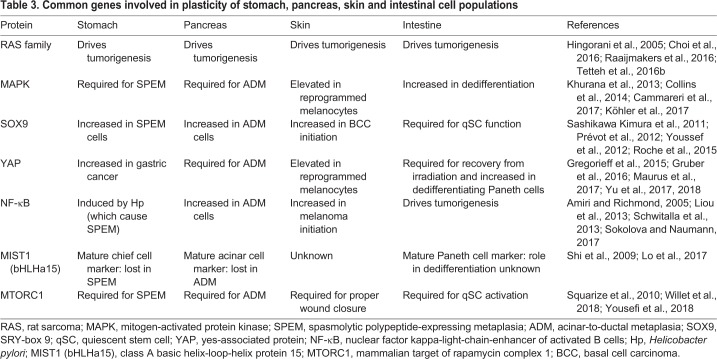
**Common genes involved in plasticity of stomach, pancreas, skin and intestinal cell populations**

Despite the current lack of consensus about how often mature cells revert to progenitors in skin (outside of the earlier studies before genetic lineage tracing and in the case of melanoma), recent studies indicate that the skin is not exceptional in what seems to be a universal tissue property. Specifically, *in vivo* live-imaging data from the Watt lab support that dedifferentiation of suprabasal cells may occur in the IFE (Donati et al., 2017). Also, even phenotypically healthy aged skin appears to be a reservoir for cancer driver mutations (Martincorena et al., 2015). This supports the possibility that plasticity of cells of any differentiation state may unlock dysplasia-causing mutations, as proposed in the latter stages of the cyclical hit model (Fig. 1). SC lineage plasticity is also seen upon ablation of specific populations of HF SCs, theoretically allowing mutations that have accumulated in one population of SCs to then expand to another compartment following wounding, as the replacement SCs bring their genome into the new SC niche. In short, there is evidence for various kinds of cell plasticity in the skin, but which types occur frequently and how they contribute to tumorigenesis is certainly still an open question. It is becoming clearer every day that tumorigenesis in other organs involves mature cells being called back into a progenitor role, which should provide an impetus to continue to investigate this type of plasticity in skin.

In the present two-part Review, we have largely focused on cell-autonomous events: cycles of paligenosis and redifferentiation leading to cells that accumulate mutations that then drive those same cells to become tumorigenic. On the other hand, plasticity might intersect with tumorigenesis in non-cell-autonomous ways. It is clear that benign neighbors in the skin and intestine can expel potential cancer-forming cell clones (Brown et al., 2017; Kon et al., 2017). It is possible that cycles of plasticity and mutations might affect niche cells such that accumulated mutations block their ability to stop malignant cells from expanding. In other words, the ‘final hit’ mutation may not occur in a tumor's cell of origin but rather in the surrounding niche cells that have otherwise constantly been suppressing the expansion of the tumorigenic cell (Burclaff and Mills, 2017). For example, deletion of *Tgfbr1* can replace the requirement for tissue wounding to initiate tumors from bulge HF SCs primed with RAS pathway mutations (Cammareri et al., 2016). It is possible that, if cells surrounding a clone acquire mutations that disrupt TGFβ signaling, then the clone that already harbors RAS pathway mutations may be able to initiate a tumor without any additional mutation load. In light of this ‘neighborhood watch’ mechanism of benign cells holding tumorigenic cells at bay, one might also suppose that tumorigenesis might depend not only on the tumor-initiating cells acquiring driver mutations in genes such as Ras, but also on their acquiring mutations that allow them to escape the vigilance of surrounding cells (Burclaff and Mills, 2017).

It is interesting to contemplate that, if plasticity of mature cells is indeed a key shared aspect of tumorigenesis, there may be opportunities to inhibit tumor initiation at the cell of origin for adult-onset cancers in multiple organs. Of course, we are just beginning to map the landscape of the possible conserved mechanisms that mediate the recruitment of mature cells back into the cell cycle. Many signaling components are shared during the plasticity events discussed in both parts of this Review (Table 2). As we continue to advance our knowledge of plasticity mechanisms in these organs, we will likely uncover additional parallels, potentially allowing for development of therapeutics to prevent or reverse tumorigenesis across many organs. Indeed, if the mechanisms governing paligenosis are conserved across tissues, cell types and species, similarly to those governing apoptosis, then our understanding of the molecular events underlying tumor cells of origin might advance relatively quickly.

Although there is an obvious call to consider roles for mature cells in cancer initiation, recent studies also suggest that we should reconsider our notions about plasticity and CSCs in established tumors. As mentioned above, the CSC model, as originally articulated over a decade ago (Clarke et al., 2006), defined CSCs as cells within tumors with the capacity to self-renew and to propagate heterogeneous lineages of cancer cells. This definition was largely based on the notions of normal tissue SCs being long-lived (label-retaining) stable populations that undergo only asymmetrical divisions resulting in unidirectional differentiation of a single daughter cell, assumptions that have been called into question (Lopez-Garcia et al., 2010; Snippert et al., 2010b). The CSC model describes some tumors as depending on a distinct cell population for their propagation, yet recent studies indicate that putative CSCs can be replaced by other tumor cells that are capable of plasticity (Merlos-Suárez et al., 2011; Junttila et al., 2015; de Sousa e Melo et al., 2017; Shimokawa et al., 2017). Rounds of dedifferentiation and reemergence of CSC attributes have also been observed in several cultured cancer cell lines (Chaffer et al., 2011; He et al., 2011). One perspective on these putative CSCs is that tumors are actually composed of plastic populations with cells that lose stemness and can be replaced by more ‘mature’ (or at least more quiescent) populations within the tumor. If cancers partly arise via reprogramming of mature cells, then it could be expected that tumors might carry a heightened propensity to reprogram (undergo paligenosis) in response to the injury caused by DNA-damaging chemotherapeutic agents or radiation. Thus, plasticity within tumors may hamper the development of targeted anti-CSC chemotherapies to induce tumor regression because those targeted CSCs may easily be replaced by other cells within the tumor. However, therapies aimed at inhibiting tumor cell paligenosis may open new avenues for treating cancer and reducing relapse.
